# Adipose tissue-derived exosomes contribute to obesity-associated liver diseases in long-term high-fat diet-fed mice, but not in short-term

**DOI:** 10.3389/fnut.2023.1162992

**Published:** 2023-05-09

**Authors:** Taesang Son, Inae Jeong, Jeongjin Park, Woojin Jun, Andre Kim, Ok-Kyung Kim

**Affiliations:** ^1^Division of Food and Nutrition, Chonnam National University, Gwangju, Republic of Korea; ^2^Human Ecology Research Institute, Chonnam National University, Gwangju, Republic of Korea; ^3^Department of Pharmaceutical Engineering, Silla University, Busan, Republic of Korea

**Keywords:** exosome, obesity, insulin resistance, high-fat diet, lipogenesis

## Abstract

**Introduction:**

Our study aimed to investigate the changes in hepatic endoplasmic reticulum (ER) stress, inflammation, insulin signaling, and lipid metabolism during the administration of a high-fat diet (HFD) in mice in order to identify correlations between obesity and metabolic disease development in the liver.

**Methods:**

We used short-, medium-, and long-term HFD periods, corresponding to 4, 8, and 12 weeks, respectively, and isolated exosomes from adipose tissue. We confirmed the effect of adipose tissue-derived exosomes on metabolic disorders in obesity in alpha mouse liver 12 (AML12) hepatocytes.

**Results:**

Adipose tissue-derived exosomes from HFD mice did not affect the AML12 cells after 4 weeks, but ER stress, inflammatory response, insulin resistance, and lipid synthesis were observed after 8 and 12 weeks. Furthermore, we confirmed that an HFD increases the amount of adipose tissue-derived exosomes in mice. Consequently, we can infer that adipose tissue-derived exosomes from HFD-fed mice significantly increase ER stress, inflammatory response, insulin resistance, and lipid synthesis in AML12 cells.

**Discussion:**

Our results demonstrate that obesity alters the effects of adipose tissue-derived exosomes in the liver, potentially becoming a risk factor in the development of obesity-induced liver diseases.

## 1. Introduction

Obesity, which can be defined as a state in which excess energy in the body is accumulated in the form of fat due to excessive food intake and lack of exercise, is a global health crisis that is becoming increasingly prevalent (1, 2). Obesity has significant adverse effects on the health of the body, with oxidative stress, endoplasmic reticulum (ER) stress, inflammation, mitochondrial dysfunction, and lipotoxicity more prominent in obese individuals in particular (3–5). These metabolic disorders can cause type 2 diabetes mellitus (T2DM), non-alcoholic fatty liver disease (NAFLD), and metabolic syndrome, among others (6, 7).

The liver plays a major role in regulating lipid metabolism. In obese individuals, NAFLD can develop due to a decrease in the liver’s ability to metabolize lipids. In obese adipose tissue, free fatty acid secretion increases along with insulin levels in the blood, thereby increasing the synthesis of free fatty acids in the liver. Collectively, these changes increase triglyceride (TG) accumulation in the liver and induce NAFLD (8, 9). In addition, inflammatory responses, ER stress, and lipotoxicity can occur in the liver, which can lead to NAFLD developing into non-alcoholic steatohepatitis (NASH), liver cirrhosis, and hepatocellular carcinoma. The latter two conditions are irreversible meaning that early intervention is essential. Studies have reported several mechanisms that can explain the pathogenesis of obesity-induced liver disease, including ER stress, inflammation, insulin resistance, and lipotoxicity (10–13) although the underlying mechanisms are still unclear.

However, numerous studies have recently demonstrated that exosomes can affect the physiological conditions of the recipient cell by playing an important role in cell-to-cell signaling (14). Exosomes, which are vesicles 30–150 nm in size, are secreted from viable cells following the fusion of multivesicular bodies (MVBs) to the plasma membrane. Exosomes contain substances such as mRNA, microRNA (miRNA), and proteins. The secreted exosomes can cause physiological and pathological changes in the recipient cell such as intercellular signal transduction by receptor-ligand binding, directly fusing with the receptor cell membrane, and inducing phagocytosis (15). Most cells in the body release exosomes, but their composition differs depending on the cell type and the physiological conditions (15, 16).

In this study, we investigated the changes in hepatic ER stress, inflammation, insulin signaling, and lipid metabolism in mice fed on a high-fat diet (HFD). We also isolated exosomes from adipose tissue according to the duration of the HFD and investigated the effect of adipose tissue-derived exosomes according to the degree of obesity on metabolic disorder in obesity in AML12 mouse hepatocytes. In this way, we aimed to determine whether there was any correlation between obesity and metabolic disease development in the liver.

## 2. Materials and methods

### 2.1. Animals

Five-week-old male C57BL6/J mice were purchased from G Biologics (Seongnam, Korea), and were fed in a controlled environment at 22–25°C with a 12 h light/dark cycle. All the mice were acclimated for 1 week before the experiment and were then subjected to either a normal or high-fat diet (HFD: diets containing 60% kcal fat) for 4–12 weeks. The animal study protocol was approved by the Institutional Animal Care and Use Committee of Chonnam National University (CNU IACUC-YB-2021-122), and the animals were fed per the Guidelines for Animal Experiments established by the university.

### 2.2. Histological observation

The mouse’s hepatic and adipose tissues were fixed overnight in a 10% neutral-buffered formaldehyde solution, rinsed with phosphate-buffered saline (PBS), and subsequently embedded in paraffin before staining with hematoxylin and eosin. The resulting sections were then examined under a light microscope.

### 2.3. Serum biochemical analysis

Blood was collected in a tube containing heparin (Sigma-Aldrich, United States) via the inferior vena cava after the mice were euthanized. The collected blood was centrifuged for 10 min at 1,000 × *g* and 4°C, and the supernatant was used for the serum assay. Alanine aminotransferase (ALT) (BioVision, Milpitas, CA, United States), glucose, TG, total cholesterol (TC), high-density lipoprotein (HDL), and low-density lipoprotein levels (LDL) (BIOMAX, Seoul, Korea) were measured in accordance with the respective manufacturer’s instructions.

### 2.4. Isolation and quantification of adipose tissue-derived exosomes

Isolation of exosomes from adipose tissues was performed following the methods described by Wei et al. (17) and Yu et al. (18). Epididymal white adipose tissue (eWAT), visceral white adipose tissue (vWAT), and subcutaneous white adipose tissue (sWAT) from mice were excised and cut into small pieces (5 mm × 5 mm). The shredded tissue was cultured in Dulbecco’s Modified Eagle Medium (DMEM) containing 10% exofree-fetal bovine serum (FBS) (2 mL media/g adipose tissue), and the culture supernatant was collected after 48 h. The supernatant was then centrifuged at 500 × *g* for 5 min, then 2,500 × *g* for 15 min, at a temperature of 4°C to remove large debris and dead cells. Exosomes were separated from the resulting supernatant using Exoquick-TC™ precipitation reagent (System Biosciences, Mountain View, CA, United States), per the manufacturer’s instructions. The resulting exosome pellet was resuspended in PBS for application purposes. The identification and quantification of the exosomes were performed using a NanoSight LM10 system (Malvern Panalytical Ltd., Malvern, UK), while the exosomal protein content was evaluated using the Bradford protein assay.

### 2.5. AML-12 cell culture

AML-12 cells (ATCC, VA, United States) were cultured in Dulbecco’s modified Eagle medium (DMEM, high glucose, Gibco, Grand Island, NY, United States), supplemented with 10% FBS (Gibco™), 1% penicillin-streptomycin (Gibco™), 1% Insulin-Transferrin-Selenium (ITS-G; Gibco™), and 40 ng/mL dexamethasone. The cells were then incubated at 37°C in a 5% CO_2_ atmosphere.

### 2.6. Exosome uptake assay

The isolated exosomes were labeled using the ExoGlow-Protein EV Labeling Kit (System Biosciences, Mountain View, CA, United States) and were incubated with the alpha mouse liver 12 (AML12) cells for 48 h. Following incubation, the cells were washed twice with PBS. The PBS was then aspirated, and the cells were fixed with 4% paraformaldehyde (PFA) for 15 min. Next, the cells were aspirated, washed twice with PBS, and permeabilized using 0.3% TritonX-100 for 1 h at 4°C. Nuclei were labeled using ProLong™ Gold Antifade Mountant with DAPI (Invitrogen, Thermo Fisher Scientific, Carlsbad, CA, United States). The slides were then covered with coverslips and observed under a fluorescence microscope (Olympus, Tokyo, Japan).

### 2.7. Western blotting

Proteins were extracted from the exosomes, liver tissue, and AML12 cells using a RIPA lysis buffer (Rockland Immunochemicals Inc., PA, United States) that included protease and phosphatase inhibitors (Thermo Fisher Scientific). First, the proteins were quantified using the Bradford protein assay (Bio-Rad Laboratories, Hercules, CA, United States), then 50–100 μg of protein were separated using 10% Mini-PROTEAN^®^ TGX™ Precast Protein Gel (Bio-Rad Laboratories, Hercules, CA, United States) and electrotransferred onto polyvinylidene difluoride (PVDF) membranes (Bio-Rad Laboratories, Hercules, CA, United States). The membranes were blocked using EveryBlot blocking buffer (Bio-Rad Laboratories, Hercules, CA, United States) for 5 min at room temperature (RT) and were then incubated with primary antibodies against CD63, IRE1a (Invitrogen), p-IRE1a (LSBio, Seattle, WA, United States), PI3K p85, p-PI3K, Akt, p-Akt, β-actin, acetyl-CoA carboxylase (ACC), p-ACC, fatty acid synthesis (FAS), and XBP1s (Cell Signaling, MA, United States) for 12 h at 4°C. Subsequently, the membranes were incubated with a secondary antibody (Anti-Rabbit IgG-HRP, Bio-Rad Laboratories, Hercules, CA, United States) for 1 h at RT. Protein levels were determined using enhanced chemiluminescence (Bio-Rad Laboratories, Hercules, CA, United States) and ChemiDoc XRS + (Bio-Rad Laboratories, Hercules, CA, United States).

### 2.8. RNA isolation and RT-PCR

RNA was extracted from the hepatic tissue and AML12 cells using the RNeasy mini kit (Qiagen, Germany). RNA was quantified using NanoDrop (Quawell Technology, CA, United States), and 100 ng of RNA was used to synthesize the complementary DNA (cDNA) using the iScript cDNA synthesis kit (Bio-Rad, CA, United States). The custom-designed primers (Table 1) and iQ SYBR green supermix (Bio-Rad, CA, United States) were added to the cDNA, and a real-time-polymerase chain reaction (RT-PCR) was performed using the CFX96Touch real-time PCR detection system (Bio-Rad, CA, United States).

**TABLE 1:** Primer sequences used in real-time PCR quantification of mRNA.

Gene	Primer sequences
CHOP	F: 5′-TCA CCT CCT GTC TGT CTC TC-3′
	R: 5′-TCT ACC CTC AGT CCT CTC CT-3′
Bip	F: 5′-AGC CAT CCC GTG GCA TAA -3′
	R: 5′-GGA CAG CGG CAC CAT AGG-3′
ATF6	F: 5′-GGC CAG ACT GTT TTG CTC TC-3′
	R: 5′-CCC ATA CTT CTG GTG GCA CT-3′
IL-β	F: 5′-GCC ACC TTT TGA CAG TGA TGA G-3′
	R: 5′-ATC AGG ACA GCC CAG GTC AA-3′
IL-6	F: 5′-CCA AGA GAT AAG CTG GAG TCA C-3′
	R: 5′-GCA CTA GGT TTG CCG AGT AGA-3′
TNF-a	F: 5′-AAG TTC CCA AAT GGC CTC CC-3′
	R: 5′-TTT GCT ACG ACG TGG GCT AC-3′
FAS	F: 5′-ACT GCC TTC GGT TCA GTC TC-3′
	R: 5′-CAC CCT CCA AGG AGT CTC AC-3′
SREBP1c	F: 5′-TGA AGA CAG ATG CAG GAG CC-3′
	R: 5′-ATG GTC CCT CCA CTC ACC A-3′
SCD1	F: 5′-GAG TAG CTG AGC TTT GGG CT-3′
	R: 5′-CAC CCC GAT AGC AAT ATC CAG T-3′
C/EBP	F: 5′-GAC AAG CTG AGC GAC GAG TA-3′
	R: 5′-GTC AGC TCC AGC ACC TTG T-3′

### 2.9. Statistical analysis

The mean ± standard deviation (SD) was used to present all data. To analyze the data, a one-way Duncan’s multiple range test was performed, followed by a one-way analysis of variance (ANOVA) or Student’s *t*-test for a two-sample comparison, utilizing SPSS statistics software (SPSS PASW Statistic 23.0, SPSS Inc., Chicago, IL, United States). Statistical significance was determined as *p* < 0.05.

## 3. Results

### 3.1. A high-fat diet induced obesity in mice after 8 weeks

**FIGURE 1:**
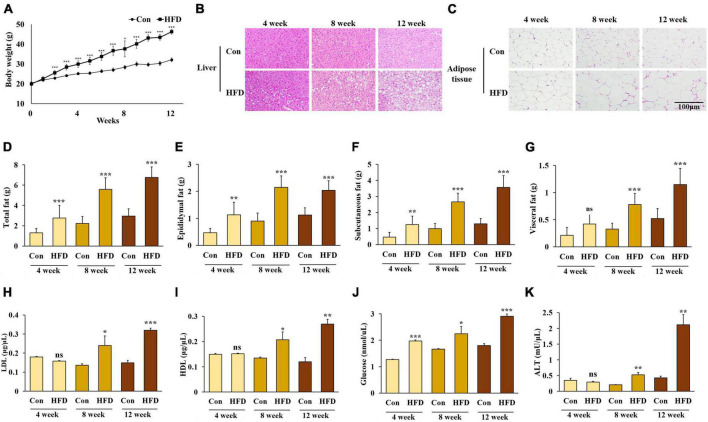
A high-fat diet induced obesity in mice. Changes in body weight **(A)**, lipid accumulation of liver **(B)** and adipose tissue **(C)**, weight of total white adipose tissue **(D)**, epididymal white adipose tissue **(E)**, subcutaneous white adipose tissue **(F)**, and visceral white adipose tissue **(G)**, and serum levels of LDL-cholesterol **(H)**, HDL-cholesterol **(I)**, triglyceride **(J)**, and alanine aminotransferase **(K)** in the HFD-fed mice. All data are expressed as mean ± SD. Con (normal diet-fed mice versus HFD-fed mice), *p < 0.05, **p < 0.01, ***p < 0.001. ns, not significant.

From the second week, the body weight of the HFD-fed mice was significantly higher than the mice on a normal diet (Figure 1A). To confirm the lipid accumulation caused by HFD, we performed histological observations of hematoxylin-eosin-stained hepatic and adipose tissues. As expected, lipid accumulation had increased in the HFD-fed mice compared with those fed a normal diet over the study period (Figures 1B, C). These results indicate that the HFD caused obesity in mice. Compared with the mice fed a normal diet, the HFD-fed mice displayed a significant increase in total white adipose tissue, eWAT, and sWAT after 4 weeks, and vWAT after 8 weeks (Figures 1D–G) (*p* < 0.05).

No significant differences were found in the levels of serum LDL, HDL, TG, and ALT between the normal diet-fed mice and the HFD-fed mice after 4 weeks. However, the levels of serum LDL, HDL, TG, and ALT were significantly higher in the HFD-fed mice after 8 weeks (Figures 1H–K) (*p* < 0.05).

### 3.2. A high-fat diet induced endoplasmic reticulum stress and an inflammatory response in hepatic tissue after 8 weeks

**FIGURE 2:**
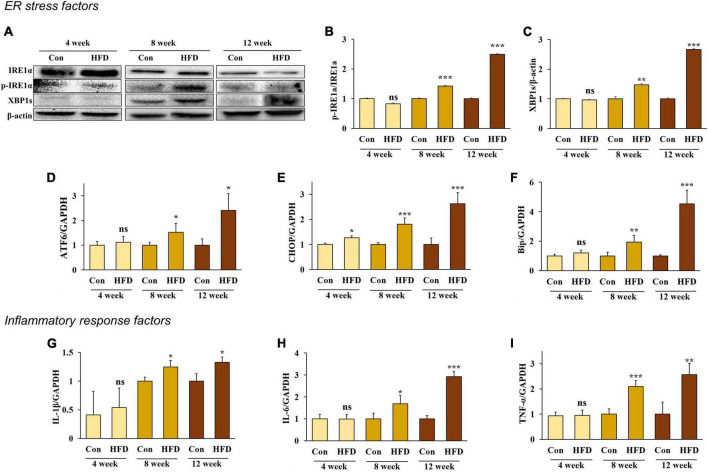
A high-fat diet induced endoplasmic reticulum stress and inflammatory response in the hepatic tissue after 8 weeks. Protein expression of IRE-1α **(A)** band, **(B)** and XBP1s **(A)** band, **(C)** and the relative mRNA expression levels of ATF6 **(D)**, CHOP **(E)**, Bip **(F)**, IL-1β **(G)**, IL-6 **(H)**, and TNF-α **(I)** in the livers of HFD-fed mice. All data are expressed as mean ± SD. Con (normal diet-fed mice versus HFD-fed mice), *p < 0.05, **p < 0.01, ***p < 0.001. ns, not significant.

The ER is an important organelle that is responsible for protein and lipid synthesis and signal transduction. Multiple studies have shown that excessive energy intake can induce ER stress in the liver (19, 20). During the feeding period, we measured the protein expression of inositol-requiring enzyme 1 α (IRE1α) phosphorylation and spliced X-box binding protein 1 (XBP1s)—both markers of ER stress—to confirm whether ER stress was present in HFD-fed mice compared with normal diet-fed mice. After 8 weeks, increased protein expression of IRE1a phosphorylation and XBP1s was confirmed in the HFD-fed mice compared with the normal diet-fed mice (Figures 2A–C).

In addition, the RNA expression of the ER chaperone protein Bip, the membrane sensor protein ATF6, and C/EBP homologous protein (CHOP), were all significantly increased in HFD-fed mice compared with the normal diet-fed mice (Figures 2D–F) (*p* < 0.05). These results indicate that an HFD can induce ER stress in the livers of mice after 8 weeks.

Previous studies have shown that hepatic inflammation is associated with obesity-induced liver disease (6). Our results confirmed that an HFD stimulates an inflammatory response and the expression of pro-inflammatory cytokines IL-1β, IL-6, and TNF-α in the liver. Our results also confirmed that the mRNA expression of IL-1β, IL-6, and TNF-α significantly increased in HFD-fed mice after 8 weeks compared with the conventional diet-fed mice (Figures 2G–I) (*p* < 0.05), indicating that an HFD can cause inflammation in the liver of mice after 8 weeks.

### 3.3. A high-fat diet induced insulin resistance and lipogenesis in hepatic tissue after 8 weeks

**FIGURE 3:**
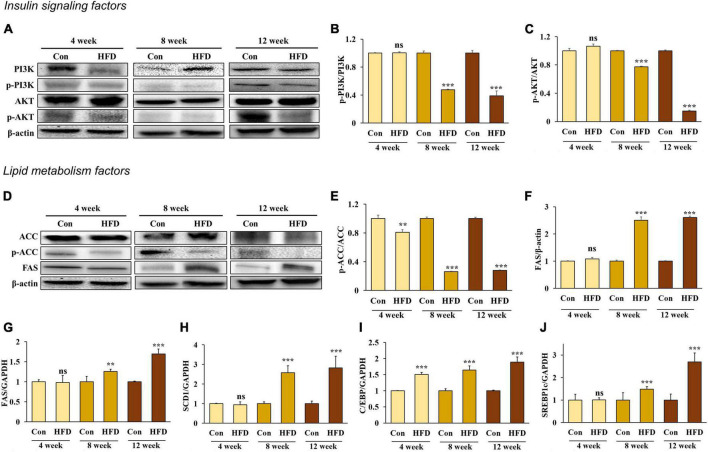
A high-fat diet induced insulin resistance and lipogenesis in the hepatic tissue after 8 weeks. Protein expression of PI3K **(A)** band, **(B)**, AKT **(A)** band, **(C)**, ACC **(D)** band, **(E)**, and FAS **(D)** band, **(F)** and relative mRNA expression levels of FAS **(G)**, SCD1 **(H)**, C/EBP **(I)**, and SREBP1c **(J)** in the livers of HFD-fed mice. All data are expressed as mean ± SD. Con (normal diet-fed mice versus HFD-fed mice), **p < 0.01, ***p < 0.001. ns, not significant.

Insulin binds to the insulin receptor (IR) of the cell membrane and phosphorylated insulin receptor substrate 1 (IRS1), activating phosphatidylinositol 3-kinases (PI3K). The activated PI3K subsequently induces protein kinase B (Akt) phosphorylation, which inhibits glycogen synthase kinase 3 and Forkhead box protein O1 and activates the mammalian target of rapamycin complex 1 to stimulate glycogen synthesis, gluconeogenesis, and lipid synthesis (21). To determine whether an HFD would induce insulin resistance in hepatic tissue, we measured the phosphorylation rate of both PI3K and AKT. The results showed that PI3K and AKT phosphorylation decreased in the hepatic tissue of the HFD-fed mice after 8 and 12 weeks (Figures 3A–C), indicating that an HFD can induce insulin resistance after 8 weeks.

The liver and adipose tissue are involved in regulating metabolic homeostasis through lipid metabolism. However, excessive hepatic lipogenesis can lead to the development of fatty liver disease. We compared the mRNA expression of adipogenesis in the liver along with the following lipogenesis factors: ACC, FAS, and stearoyl-CoA desaturase-1 (SCD1), CCAAT/enhancer binding protein (C/EBP), and sterol regulatory element binding protein-1c (SREBP1c). We found that ACC activation (Figures 3D, E) and the mRNA expression of C/EBP (Figure 3I) increased significantly in the HFD-fed mice compared with those in normal diet-fed mice, starting from week 4 and further increasing after 8 and 12 weeks. In addition, FAS (Figures 3F, G), SCD1 (Figure 3H), and SERBP1c (Figure 3J) expression increased significantly in the HFD-fed mice compared with the normal diet-fed mice from 8 weeks onward (*p* < 0.05), suggesting that an HFD induces TG accumulation in the liver via lipogenesis from 8 weeks onward.

### 3.4. A high-fat diet stimulated exosome secretion from the adipose tissue after 8 weeks

**FIGURE 4:**
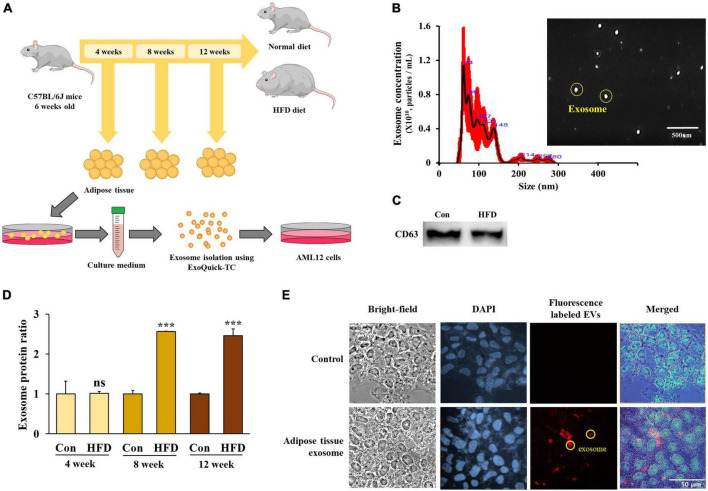
The high-fat diet stimulated exosome secretion from the adipose tissue after 8 weeks. Experimental schematic of exosome isolation from mouse adipose tissue and exosome treatment in AML12 cells **(A)**, exosome size and vesicle observation using NanoSight **(B)**, protein levels of CD63 from isolated exosomes **(C)**, protein amounts of adipose tissue-derived exosomes from total eWAT/mouse **(D)**, and red fluorescence-labeled adipose tissue-derived exosome uptake in AML12 cells **(E)**. All data are expressed as mean ± SD. Con (normal diet-fed mice versus HFD-fed mice), ***p < 0.001. ns, not significant.

Exosomes were extracted from adipose tissue (Figure 4A), and their size and expression of the exosome marker CD63 were measured. The size distribution of the extracted vesicles was found to have a predominant peak between 40–150 nm, which is consistent with the typical size range of exosomes (Figure 4B). Additionally, we confirmed the presence of CD63 expression in the isolated exosomes from adipose tissue (Figure 4C), thereby confirming their identity as exosomes.

In order to quantify the adipose tissue-derived exosomes, we evaluated the total protein expression level after the vesicles had lysed. After 4 weeks, there was no difference between the HFD-fed mice and normal diet-fed mice in the total protein content of the eWAT. However, exosome secretion increased significantly in the HFD-fed mice after 8 and 12 weeks compared with the normal diet-fed mice (Figure 4D) (*p* < 0.001), suggesting that the secretion of adipose tissue-derived exosomes increased in HFD-fed mice after 8 weeks.

In order to exert a physiological effect, adipose tissue-derived exosomes must be taken up by the recipient cells. Therefore, we tagged a PKH-26 red fluorescent dye to the adipose tissue-derived exosomes before adding them to the culture medium of the AML12 cells to observe the extent of exosome uptake.

After 48 h of treatment, fluorescence was observed in the AML12 cells (Figure 4E) confirming that adipose tissue-derived exosomes can be taken up into AML12 cells and suggesting that exosomes play a potential role in the induction of specific physiological changes within the cells.

### 3.5. Adipose tissue-derived exosomes from high-fat diet-fed mice induced endoplasmic reticulum stress and an inflammatory response in AML12 cells

**FIGURE 5:**
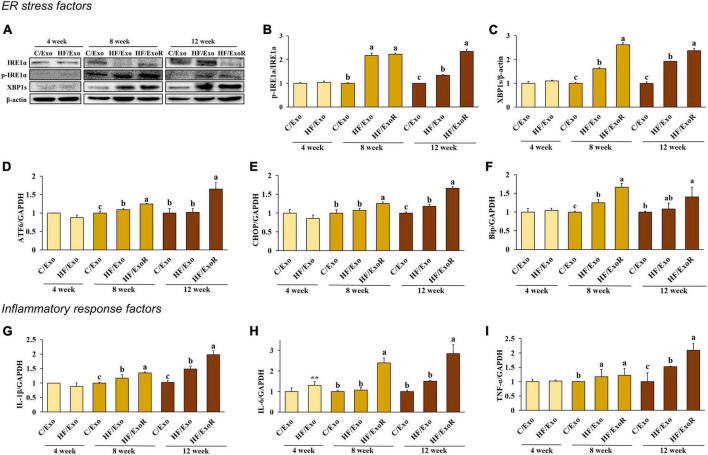
Adipose tissue-derived exosomes from high-fat-diet-fed mice induced endoplasmic reticulum stress and inflammatory response in AML12 cells. Protein expression of IRE-1α **(A)** band, **(B)** and XBP1s **(A)** band, **(C)** and relative mRNA expression levels of ATF6 **(D)**, CHOP **(E)**, Bip **(F)**, IL-1β **(G)**, IL-6 **(H)**, and TNF-α **(I)** in AML12 cells treated with adipose tissue-derived exosomes from normal diet mice (C/Exo, 50 μg/mL), HFD mice (HF/Exo, 50 μg/mL), or differential ratio-based adipose tissue-derived exosomes from HFD mice (HF/ExoR, 125 μg/mL) over 48 h. All data are expressed as mean ± SD. 4 week: C/Exo versus HF/Exo, **p < 0.01. 8 and 12 weeks: Different letters indicate a significant difference, *p* < 0.05 (a > b > c), as determined by Duncan’s multiple range test.

In the previous experiment, we determined that ER stress was induced in the liver of HFD-fed mice. To determine whether adipose tissue-derived exosomes directly induced ER stress, we investigated the ER stress factors in adipose tissue-derived exosome-treated AML12 cells. Cells were treated with 50 μg/mL of adipose tissue-derived exosomes from normal diet-fed mice (C/Exo), 50 μg/mL from HFD-fed mice (HF/Exo), and a further 125 μg/mL from HFD-fed mice obtained from the differential ratio to normalize to the weight of the adipose tissue (HF/ExoR). Interestingly, we found that HF/Exo and HF/ExoR induced ER stress after 8 and 12 weeks, with the HF/ExoR treatment significantly increasing ER stress (Figures 5A–F). In particular, the HF/ExoR group showed a dramatic increase in the ER stress factors.

To determine whether adipose tissue-derived exosomes directly induce liver inflammation, we measured the pro-inflammatory cytokines in adipose tissue-derived exosome-treated AML12 cells. Figures 5G–I shows that treatment with HF/Exo and HF/ExoR significantly increased the mRNA expression of pro-inflammatory cytokines in AML12 cells compared with the C/Exo treatment after 8 and 12 weeks (*p* < 0.05), indicating that adipose tissue-derived exosomes cause inflammation in the livers of mice after 8 weeks of an HFD.

### 3.6. Adipose tissue-derived exosomes from high-fat diet-fed mice induce insulin resistance and lipogenesis in AML12 cells

**FIGURE 6:**
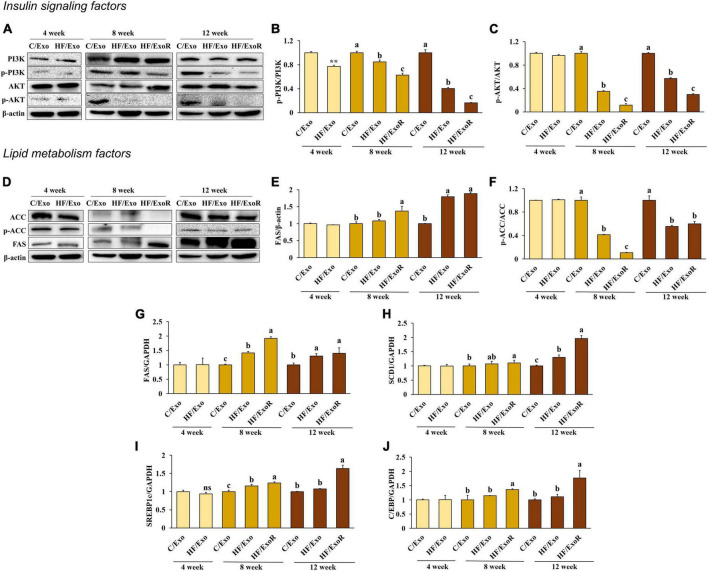
Adipose tissue-derived exosomes from high-fat-diet-fed mice increased insulin resistance and lipogenesis levels in AML12 cells. Protein expression of PI3K **(A)** band, **(B)**, AKT **(A)** band, **(C)**, ACC **(D)** band, **(E)**, and FAS **(D)** band, **(F)** and relative mRNA expression levels of FAS **(G)**, SCD1 **(H)**, C/EBP **(I)**, and SREBP1c **(J)** in AML12 cells treated with adipose tissue-derived exosomes from normal diet mice (C/Exo, 50 μg/mL), HFD mice (HF/Exo, 50 μg/mL), or differential ratio-based adipose tissue-derived exosomes from HFD mice (HExoR, 125 μg/mL) over 48 h. All data are expressed as mean ± SD. 4 week: C/Exo versus HF/Exo, **p < 0.01. 8 and 12 weeks: Different letters indicate a significant difference, *p* < 0.05 (a > b > c), as determined by Duncan’s multiple range test. ns, not significant.

We measured the insulin signaling pathway in adipose tissue-derived exosome-treated AML12 cells to determine whether obesity would affect this pathway. Compared with the C/Exo treatment, we found that AKT phosphorylation decreased significantly with the HF/Exo treatment from week 4, while PI3K phosphorylation decreased significantly from week 8. In addition, the decrease in AKT and PI3K phosphorylation was more significant in the HF/ExoR treatment (Figures 6A–C) indicating that adipose tissue-derived exosomes in the liver of mice develop insulin resistance after 8 weeks of an HFD.

We hypothesized that lipogenesis in the livers of the HFD-fed mice was caused by adipose tissue-derived exosomes. From week 8, we found that ACC activation and FAS increased significantly in the HF/Exo and HF/ExoR treatments compared with the C/Exo treated cells (Figures 6D–F). In addition, the mRNA expression levels of FAS, SERBP1c, SCD1, and C/EBP increased significantly with the HF/ExoR treatment when compared with the C/Exo treatment (Figures 6G–J) (*p* < 0.05). Our results suggest, therefore, that adipose tissue-derived exosomes from HFD mice can stimulate TG accumulation in the liver as a consequence of lipogenesis.

## 4. Discussion

Multiple metabolic processes such as glycolysis, gluconeogenesis, lipolysis, and lipogenesis take place in the liver, which also regulates physical homeostasis by releasing insulin, glucagon, growth hormones, and enzymes (22, 23). Obesity is known to cause metabolic disorders within the liver. For example, obesity-mediated hypertrophy of adipose tissue stimulates the secretion of leptin, resistin, and pro-inflammatory cytokines, which can induce ER stress, inflammation, and metabolic abnormalities in the liver (24–27). Metabolic changes in adipose tissue in obese individuals can have a significant adverse effect on hepatic metabolism, but the mechanism has not been fully elucidated thus far. Recent studies have suggested that adipose tissue-derived exosomes may play a role in this process by exerting physiological and pathological effects on various organs (28, 29). Furthermore, the internal composition of exosomes can vary depending on the state of the cells (30, 31). Therefore, in order to confirm how adipose tissue-derived exosomes affect the mechanism of obesity-mediated liver disease development, we investigated the effect of adipose tissue-derived exosomes on hepatocytes using mice fed on an HFD for varying durations.

After 4 weeks, only increases in CHOP and ACC activity were confirmed in HFD-fed mice compared with normal diet-fed mice. However, after 8 and 12 weeks, ER stress was confirmed in HFD-fed mice via an increase in IRE1a, XBP1s, ATF6, CHOP, and Bip levels, while an inflammatory response was confirmed through increases in the levels of pro-inflammatory cytokines IL-1β, IL-6, and TNF-α. In addition, the activation of PI3K and AKT in the insulin signaling pathway was inhibited, while the expression levels of FAS, ACC, SREBP1c, SCD1, and C/EBP, which are involved in lipogenesis and adipogenesis, increased. These results indicate that ER stress, inflammation, insulin resistance, and lipogenesis have been stimulated in the livers of mice after 8 weeks of an HFD.

A recent study reported that exosome secretion from adipose tissue was elevated in obese individuals (32). After quantifying adipose tissue-derived exosomes, our study found no change in the amount of adipose tissue-derived exosomes after 4 weeks of an HFD. However, we did confirm that adipose tissue-derived exosome levels increased in the HFD-fed mice compared with the normal diet-fed mice after 8 and 12 weeks. These results suggest that the amount of circulating adipose tissue-derived exosomes in the blood is higher in HFD-fed mice.

After 8 and 12 weeks, ER stress levels and inflammatory markers increased in cells treated with adipose tissue-derived exosomes from the HFD-fed mice. Simultaneously, the activation of PI3K and AKT in the insulin signaling pathway was diminished, while the expression levels of FAS and ACC were elevated. A previous study showed similar results, where a reduced level of AKT activation was caused by a reduction of the miRNA-141-3p levels in adipose tissue-derived exosomes from HFD-fed mice (33). Rong et al. (34) also noted that IRE1a and CHOP expression levels increased in the liver because of the presence of adipose tissue-derived exosomes from HFD-fed mice.

In addition, when considering the amount of circulating adipose tissue-derived exosomes in HFD-fed mice, the HF/ExoR cells showed an increase in the levels of ER stress markers at 8 and 12 weeks compared with those in the control group. Moreover, levels of pro-inflammatory cytokines, FAS, ACC, SREBP1c, SCD1, and C/EBP increased, while PI3K and AKT activation in the insulin signaling pathway decreased. Our data confirmed that increased amounts of circulating adipose tissue-derived exosomes in 8-week HFD-fed mice accelerated stimulation of ER stress, inflammation, insulin resistance, and lipid synthesis in AML12 cells. These accelerations increased further in adipose tissue-derived exosomes in the 12-week HFD-fed mice.

These results show that the effect of adipose tissue-derived exosomes worsens in proportion to the degree of obesity, suggesting that adipose-derived exosomes can function as risk factors for T2DM and NAFLD in the liver. It is hoped that the results of this study can facilitate the use of exosomes as biomarkers for disease development and disease treatment through the control of exosomes.

In our study, obesity was induced in mice by being fed a high-fat diet, and the effect of adipose tissue-derived exosomes on AML12 cells was confirmed by subdividing the obesity-induced state over the period of dietary intake. Referring to the reported protein and mRNA cargo that contribute to obesity-related liver disease (35, 36), further studies are needed to observe changes in the content of adipose tissue-derived exosomes during the high-fat diet intake period. Additionally, the correlations regarding the various effects of adipose tissue-derived exosomes on the liver are currently unclear. Additional research is necessary to measure exosome content and determine the factors affecting liver metabolism and to shed light on the molecular mechanisms triggered by adipose tissue-derived exosomes in the liver.

## 5. Conclusion

In conclusion, our study revealed the changes potentiated by the induction of obesity in mice through an HFD. Our study also determined the effect of adipose tissue-derived exosomes in hepatocytes. ER stress, inflammation, insulin resistance, and lipid synthesis increased significantly after 8 weeks of an HFD. In addition, we confirmed that adipose tissue-derived exosomes can be assimilated by AML12 cells, inducing ER stress, inflammation, insulin resistance, and lipogenesis from week 8. The effect of the treatment with adipose tissue-derived exosomes from HFD mice is relevant considering the increase in the amounts of adipose tissue-derived exosomes in obese mice. Based on these results, we confirmed that persistent excessive energy intake causes liver stress, inflammatory response, insulin resistance, and lipotoxicity, which could potentially be mediated by adipose tissue-derived exosomes. These results contribute to our understanding of the mechanisms underlying exosome function in the development of obesity-related metabolic diseases.

## Data availability statement

The original contributions presented in this study are included in the article/supplementary material, further inquiries can be directed to the corresponding authors.

## Ethics statement

The animal study protocol was approved by the Institutional Animal Care and Use Committee of Chonnam National University (CNU IACUC-YB-2021-122), and the animals were fed per the Guidelines for Animal Experiments established by the university.

## Author contributions

TS: investigation, methodology, writing, and analysis. IJ, JP, WJ, and AK: investigation and analysis. O-KK: conceptualization, methodology, writing, funding, and supervision. All authors contributed to the article and approved the submitted version.
